# Cyclization Reaction Catalyzed by Cyclodipeptide Synthases Relies on a Conserved Tyrosine Residue

**DOI:** 10.1038/s41598-018-25479-5

**Published:** 2018-05-04

**Authors:** Emmanuelle Schmitt, Gabrielle Bourgeois, Muriel Gondry, Alexey Aleksandrov

**Affiliations:** 1 Laboratoire de Biochimie (CNRS UMR7654), Department of Biology, Ecole Polytechnique, F-91128 Palaiseau, France; 2 Institute for Integrative Biology of the Cell (I2BC), CEA, CNRS, Univ. Paris-Sud, Université Paris-Saclay, 91198 Gif-sur-Yvette cedex, France

## Abstract

Cyclodipeptide synthases (CDPSs) form various cyclodipeptides from two aminoacyl tRNAs via a stepwise mechanism with the formation of a dipeptidyl enzyme intermediate. As a final step of the catalytic reaction, the dipeptidyl group undergoes intramolecular cyclization to generate the target cyclodipeptide product. In this work, we investigated the cyclization reaction in the cyclodipeptide synthase AlbC using QM/MM methods and free energy simulations. The results indicate that the catalytic Y202 residue is in its neutral protonated form, and thus, is not likely to serve as a general base during the reaction. We further demonstrate that the reaction relies on the conserved residue Y202 serving as a proton relay, and the direct proton transfer from the amino group to S37 of AlbC is unlikely. Calculations reveal that the hydroxyl group of tyrosine is more suitable for the proton transfer than hydroxyl groups of other amino acids, such as serine and threonine. Results also show that the residues E182, N40, Y178 and H203 maintain the correct conformation of the dipeptide needed for the cyclization reaction. The mechanism discovered in this work relies on the amino groups conserved among the entire CDPS family and, thus is expected to be universal among CDPSs.

## Introduction

Cyclodipeptide synthases (CDPSs) are a family of enzymes that use aminoacyl-tRNAs (aa-tRNAs) to synthetize cyclodipeptides, which are precursors of many secondary metabolites with diverse biological functions^1,2^. The first member of this family was identified in 2002 during characterization of the albonoursin biosynthetic pathway in *Streptomyces noursei* and called AlbC^3^. Three CDPSs are structurally characterized and all three share a common architecture, consisting of a monomer containing a Rossmann fold domain^4–7^. The CDPSs display strong structural similarity to the catalytic domains of class Ic aminoacyl tRNA synthetases (aaRSs), suggesting that CDPSs evolved from the class Ic of aaRSs^1^. However, there are several significant differences between CDPSs and class Ic aaRSs. The ATP-binding motifs present in aaRSs are not present in CDPSs, since there is no need to activate amino acids in CDPSs, and CDPSs are active as monomers in contrast to the TyrRS and TrpRS that function as homodimers.

The catalytic mechanism has been extensively studied experimentally for the structurally characterized CDPSs, and a structure mimicking a reaction intermediate was obtained for AlbC^7^. It was demonstrated that AlbC uses Phe-tRNA^Phe^ and Leu-tRNA^Leu^ (or a second Phe-tRNA^Phe^) as substrates for a ping-pong mechanism involving the formation of two successive acyl-enzyme intermediates^1^. The catalytic reaction starts with the binding of the first aa-tRNA and the transfer of its aminoacyl moiety to a conserved serine residue leading to the formation of an aminoacyl enzyme intermediate. For the second step, the tRNA^Phe^ part of the first substrate dissociates from AlbC and a second aa-tRNA binds to the enzyme. The phenylalanyl-AlbC reacts with the second aa-tRNA to form a dipeptidyl-AlbC intermediate. In the final step, the target cyclodipeptide is obtained through intramolecular cyclization.

Residues important for the reaction in AlbC were identified through site-directed mutagenesis and chemical biology studies^5,7^. These residues, apart from S37, the conserved residue that accepts the aminoacyl group, are Y202, Y178, E182, N40, and H203. The residues Y178 and E182 are involved in the stabilization of the aminoacyl moiety of the first substrate (named Phe1) throughout the catalytic cycle as suggested by the crystal structure of the diphenylalanyl-enzyme intermediate mimic^7^. E182 was also suggested to act as a general catalytic base during the formation of the dipeptidyl-enzyme by deprotonating the ammonium group of the aminoacyl-enzyme, enabling it to attack the carbonyl group of the second substrate to form the dipeptidyl-enzyme intermediate^4,7^. It was shown that the entire family of CDPSs can be classed into two subfamilies, so called NYH and XYP, characterized by the presence of specific sequence signatures at positions N40, Y202, and H203^1^. The residues, N40 and H203 were suggested to play a role in the stabilization of other residues in the catalytic center and are not conserved among the CDPS family, but are important for the function of AlbC^1^. In contrast to N40 and H203, the residue Y202 is strictly conserved and has been suggested to play a key role during the cyclization step of the reaction. It was demonstrated that the Y202F mutation does not affect the formation of the aminoacyl enzyme intermediate^5^, but leads to the accumulation of the covalent dipeptidyl enzyme intermediate, which was not observed with the wild-type enzyme^7^. Therefore, Y202 is involved in the cyclization process, and was proposed to deprotonate the primary amine group of the second phenylalanyl of the dipeptide (named Phe2), which is also consistent with the short distance between the hydroxyl group of Y202 and the N atom of the dipeptide N-terminal end observed in the experimental structure of the dipeptide intermediate^7^.

The cyclization reaction catalyzed by CDPSs is unique to CDPS, and absent in, aaRSs and which has never been studied by computational work before. In this work we have investigated the cyclization reaction catalyzed by AlbC using a set of simulation techniques, including free energy calculations using molecular mechanics (MM) to establish which group of the complex might participate as proton acceptor/donor, DFT hybrid quantum chemical (QM)/MM potentials together with reaction-path-finding algorithms to test possible mechanistic pathways, and QM/MM free energy calculations to determine reaction barriers.

## Results

### Protonation state of the protein

**Figure 1:**
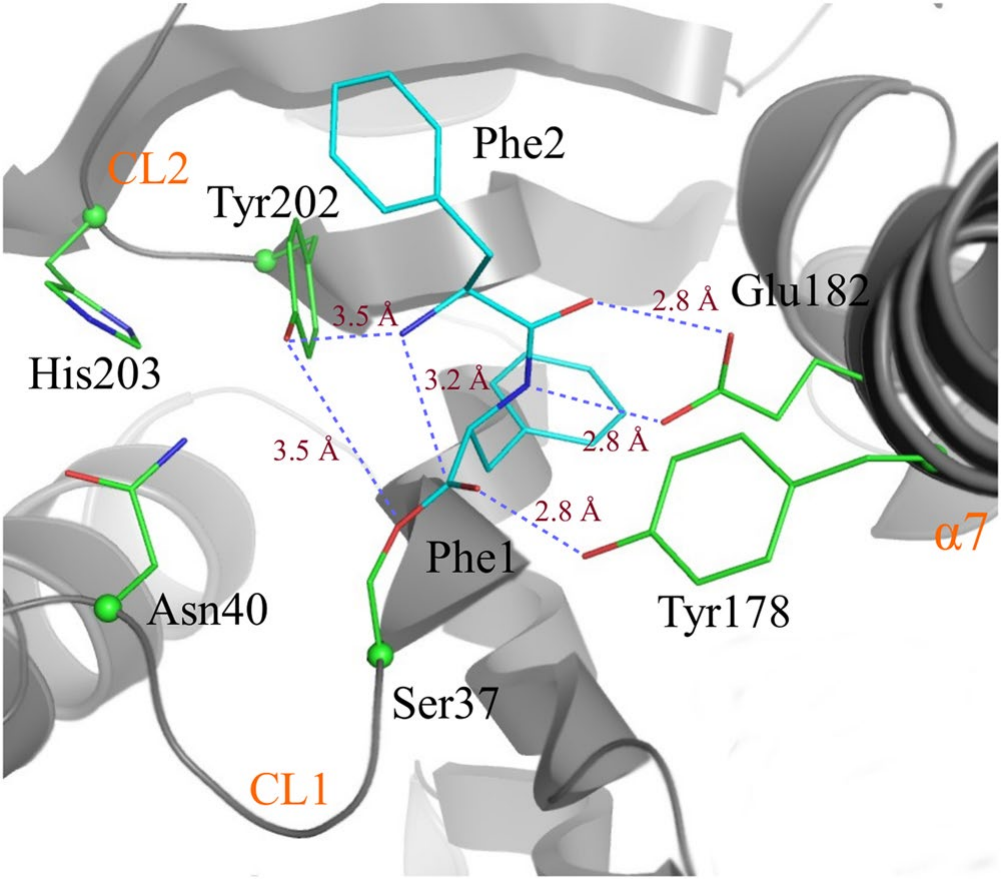
The active site pocket of the AlbC cyclodipeptide synthase. Important residues of AlbC identified through biochemical experiments are shown. The average distances observed in the MD simulations between important groups are indicated.

**Figure 2:**
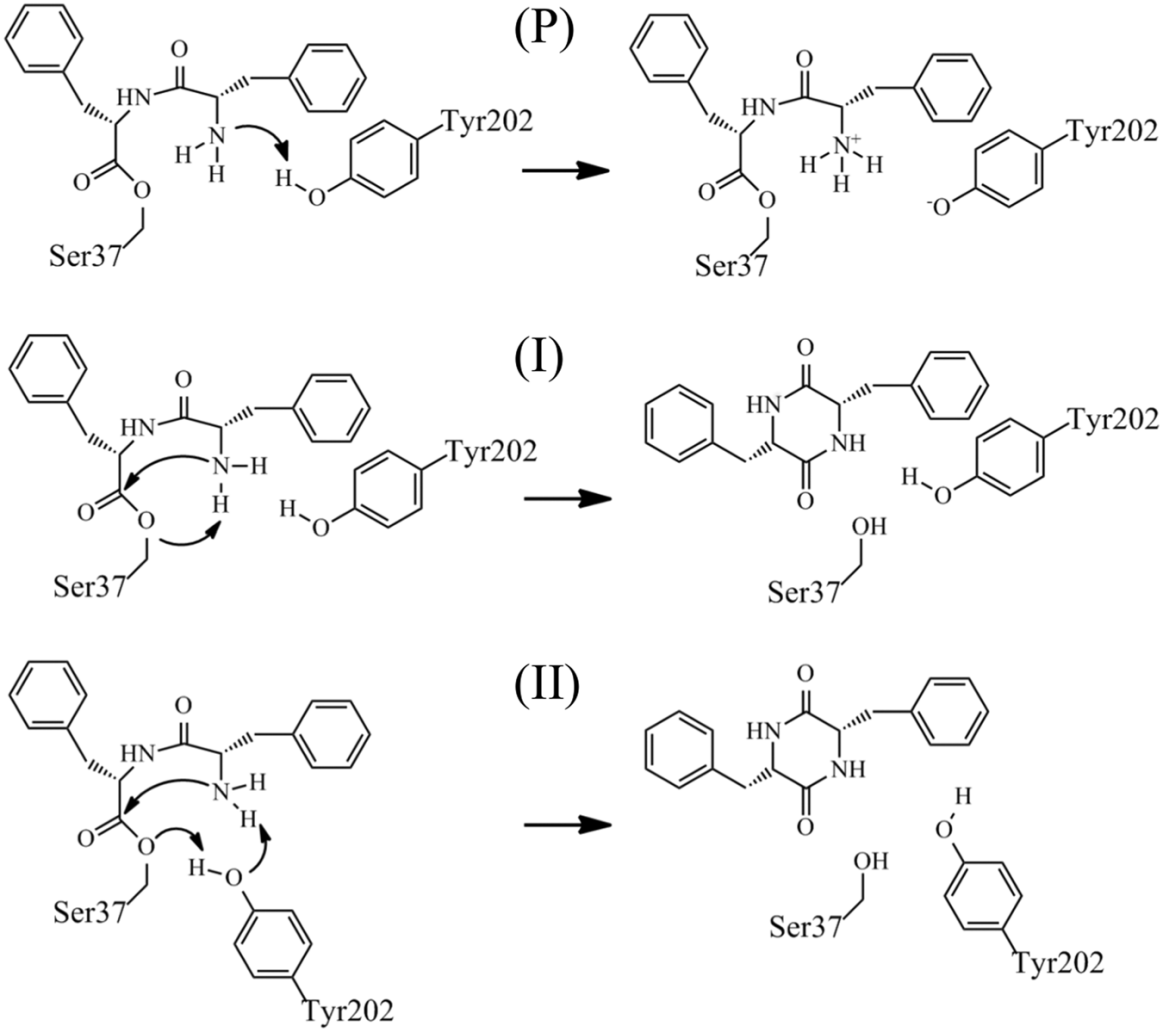
Schematics of the mechanisms investigated in the QM/MM-FEP simulations. Mechanism P corresponds to the proton transfer from Y202 to the N-terminus end of the dipeptide; mechanisms I and II correspond to the cyclization reaction (I) with the direct proton transfer and (II) using Y202 as a proton relay.

Prior to studies of mechanisms of enzyme reactions, it is necessary to establish the most favorable protonation states of the enzyme, since the charges on the residues can have a significant effect on the computed free energies. Specifically, in AlbC we are interested in the protonation state of the catalytic residue Y202 and the Phe2 amino group of the dipeptide covalently attached to S37 of AlbC. The structure of the catalytic pocket of AlbC bound to the modeled dipeptide is shown in Fig. 1. Protonation and deprotonation of these two groups in the protein give four possible states. To rank these states, we use QM/MM free energy perturbation (QM/MM FEP) simulations and the Poisson-Boltzmann/Linear Response Approximation (PB/LRA) coupled to the classical force field simulations as described in the Methods section. The computed free energies of these states are given in Table 1. Using the QM/MM FEP method we computed the free energy of the proton transfer from the hydroxyl group of Y202 to the neutral amino group. During this reaction, the proton from the hydroxyl group of Y202 shifts to the amino group as shown in Fig. 2. The computed free energy associated with the proton transfer is 2.7 ± 0.8 kcal/mol favoring the state with the neutral amino group and neutral tyrosine. The reaction is barrier-less with the gradual increase of the free energy with the proton transfer to the NH_2_ group. To test the effect of the enzyme environment on the stabilization of the neutral amino group we recomputed the free energy of the proton transfer in vacuum and in the COSMO implicit solvent^8^ using the geometry of the QM region taken from the protein simulations. The computed energy for the proton transfer from the tyrosine to the amino group is 14.5 kcal/mol and 7.1 kcal/mol in vacuum and in the implicit solvent, respectively, both favoring the state with the neutral tyrosine. Thus, the state with the charged tyrosine form is not favored in the protein and in the solvent, but the enzyme environment strongly favors the proton transfer from Y202 to the amino group by 4.4 kcal/mol relative to solvent.

**Table 1:** Relative free energies of the four protonation states of AlbC.

Protonation state		ΔG, kcal/mol
Y202	N-terminus	
OH	NH_2_	0.0
O^(−)^	NH_3_^(+)^	+2.7 (0.8)^a^
OH	NH_3_^(+)^	+0.8 (0.5)^b^
O^(−)^	NH_2_	+7.7 (0.6)^b^

The free energies are given relative to the state with the neutral protonated Y202 and the neutral deprotonated amino group. ^a^The free energy was computed using the QM/MM FEP method. ^b^The free energies were computed using the PB/LRA method. The uncertainties are given in parentheses.

Using the PB/LRA method we computed the relative free energies of the other two states. The free energy of the state with the neutral protonated tyrosine and the charged amino group relative to the state with the neutral tyrosine and the neutral deprotonated amino group is computed to be 2.9 ± 0.5 kcal/mol. In these PB/LRA simulations only the charges on the amino group were modified from the deprotonated state to the protonated state, while the tyrosine hydroxyl group was protonated. Combining this value with the pK_a_ of 8 of the N-terminal group of the model compound^9^, which is a dipeptide in solvent, we obtain using Equation 3 the absolute protonation free energy of 0.8 kcal/mol favoring the state with the neutral NH_2_ group. Thus, at physiological pH the N-terminal end group of the dipeptide prefers to be in the neutral deprotonated form. The computed free energy of the state with the charged Y202 that lost its hydroxyl hydrogen and the deprotonated amino group relative to the state with the neutral Y202 and amine group is 2.7 ± 0.6 kcal/mol. In these PB/LRA simulations only the charges on the hydroxyl group of Y202 were modified from the protonated state to the deprotonated state, while the amine group was singly protonated. In combination with the relatively high pK_a_ of the tyrosine in solvent of 10.1 pK_a_ units^10^, we obtain using Equation 3 the absolute deprotonation free energy of Y202 in the enzyme of 7.7 kcal/mol strongly favoring the state with neutral Y202. The later result can be understood since Y202 is located within a hydrophobic pocket of AlbC and does not have partners, apart from the deprotonated amino group, that could provide electrostatic stabilization of the negative charge on the tyrosine leading to the unfavorable deprotonation free energy in the protein relative to solvent. This result is an agreement with the PROPKA prediction for the pK_a_ of Y202 of 15.0 pK_a_ units. Overall, in contrast to the early hypothesis made based on the crystal structure^7^, Y202 strongly prefers to be in the protonated neutral form in AlbC. The measured pK_a_ value of tyrosine averaged over a large number of proteins was reported to be 10.3 ± 1.2 pH units^11^, which is much lower than the pK_a_ of Y202 in AlbC, suggesting that the neutral form of Y202 is needed for the catalytic function of AlbC.

### Mechanism of cyclization

We investigated the mechanism of cyclization in AlbC using the QM/MM free energy method described in the Methods section. For the reaction, the terminal amino group of the dipeptide covalently bonded to S37 should be in the deprotonated neutral form. This form in AlbC is predominant at the physiological pH as demonstrated by the free energy calculations above. During the cyclization reaction one of the two protons of the amino group should be transferred to a protein group, while the hydroxyl group of the serine residue bonded to the dipeptide should become protonated. Indeed, the pK_a_ of the hydroxyl group of serine is very high (the pK_a_ of ethanol is 16)^12^ and AlbC lacks any cationic group in the vicinity of the dipeptide, indicating that stable protein intermediates with the deprotonated species of serine would all have prohibitively high free energy relative to the reactant state. AlbC also lacks any general base and acid in the vicinity of S37 and the amine-terminus group of the dipeptide, except residue E182, which makes hydrogen bonds to the backbone of the dipeptide and, thus cannot participate in the reaction. For the reasons given above, it is more plausible that the proton should be transferred from the amino group to the hydroxyl group of S37 simultaneously with nucleophilic attack of the nitrogen atom of the dipeptide N-terminal end on the carbon atom of the dipeptidyl ester group. We tested two mechanisms, shown in Fig. 2, which differ by the way this proton is transferred: directly from the dipeptide amino group to the O atom of S37, and using Y202 serving a proton relay.

### Cyclization reaction with the direct proton transfer from the Phe2 amino group to S37

**Figure 3:**
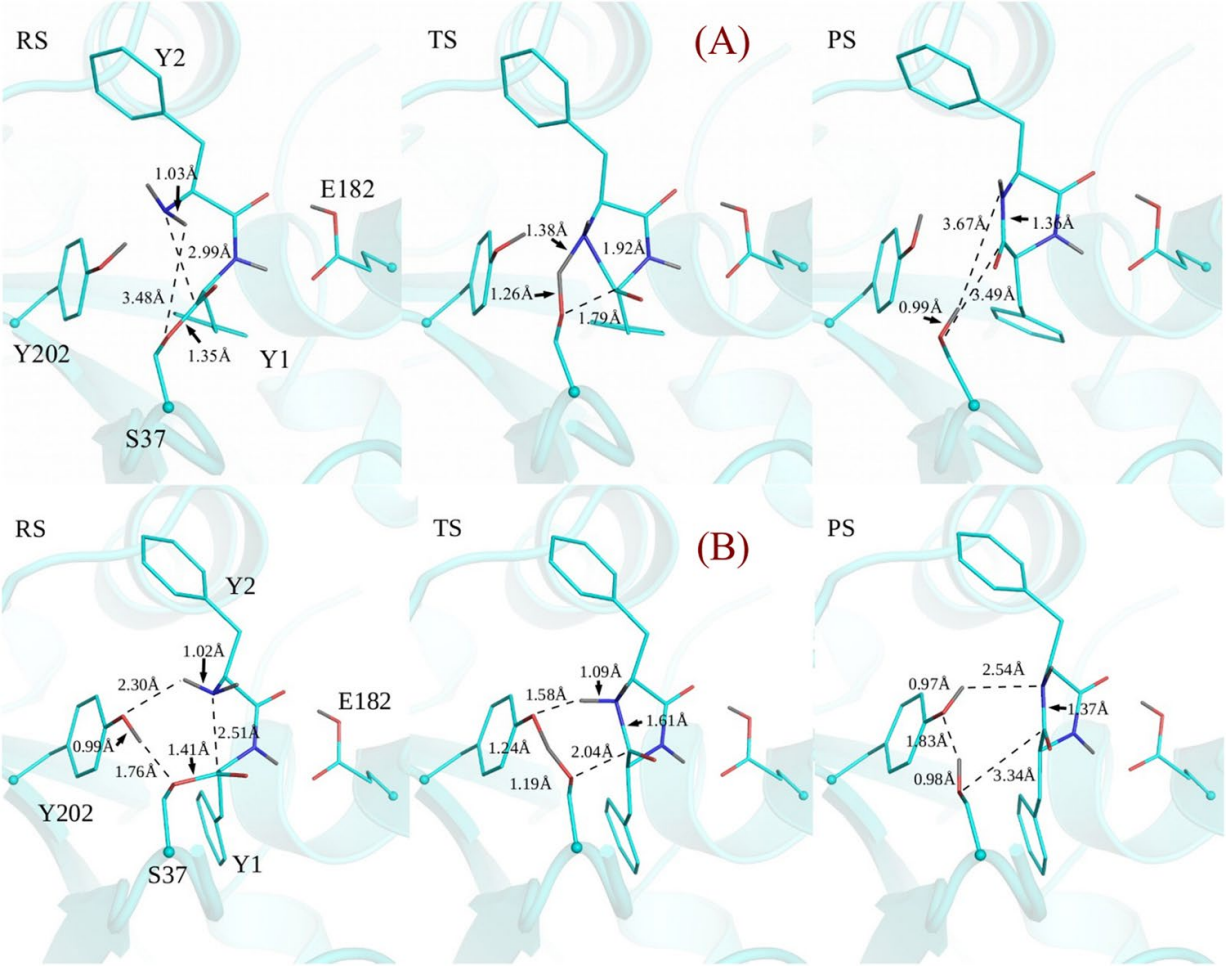
Structures on the pathway of (**A**) the self-assisted mechanism I and (**B**) mechanism II employing Y202 as a proton relay.

We investigated mechanism I shown in Fig. 2, which does not involve Y202 in the catalytic reaction. Indeed, in the MD simulations the average distance between one of the two protons of the amino group and the O atom of S37 is 2.8 Å suggesting that the proton, in principle, may be transferred directly to the hydroxyl group of S37 during the reaction. The structures of reactant (RS), transition states (TS), and products (PS) for this direct mechanism are shown in Fig. 3. The activation free energy for this mechanism was computed to be 61.6 kcal/mol, relative to the reactant state. The product is 6.1 kcal/mol lower in energy than the reactant. The free energy along the reaction pathway has a single maximum with no stable intermediates. The computed high activation energy clearly indicates that this mechanism is unlikely. In the transition state, the proton from the amino group is shared by the amino N atom and S37 with distances between the nitrogen of the amino group and the hydrogen, and the S37 oxygen and the hydrogen of 1.38 Å and 1.26 Å, respectively. The bond between the nitrogen of the amino group and the carbon of the carbonyl group is partially formed with the distance between the nitrogen and carbon atom of 1.92 Å.

To quantify the effect of the direct contribution from the enzyme environment on the reaction, we computed the energy along the reaction pathway in vacuum using the QM region geometry from the protein simulations. The transition state in vacuum was found to be lower by +7.4 kcal/mol relative to the enzyme case. Thus, for this mechanism the enzyme environment does not contribute favorably to the reaction. We also computed the energy of the transition state in the COSMO implicit solvent^8^ using the QM region from the protein simulations. With the implicit solvent the free energy of the transition state is 60.3 kcal/mol. Thus, the high energy of the transition state in this mechanism can be explained by the unfavorable contribution from the QM region only. In the TS the angles formed by the N atom of the amino group and atoms that participate in covalent interactions with the N atom are 141.9°, 108.9°, 62.9°, 108°, 106.0°, 108.3°, and 114.9°. Some of these angles strongly deviate from the angle of the ideal tetrahedral geometry of 109.5° (such as observed in tetramethylammonium), indicating that there is steric hindrance explaining the high free energy of the transition state.

### Cyclization with the participation of Y202 in the catalytic function

We also investigated the reaction with the participation of Y202 (mechanism II in Fig. 2). In this mechanism, the proton of the N-terminal amino group of the dipeptidyl group is shifted to the Y202 hydroxyl oxygen, while the proton of the hydroxyl group of Y202 moves to the oxygen of the S37 side chain in a concerted manner with a simultaneous bond formation between the N atom and carbonyl C atom. In this reaction the hydroxyl group of Y202 acts as a proton relay. The structures of reactant (RS), transition states (TS), and products (PS) for this mechanism are shown in Fig. 3. The free energy of the transition state was computed to be 12.4 ± 1.0 kcal/mol relative to reactant state. The product is −9.2 ± 1.0 kcal/mol lower than the reactant. In the TS state, the proton of the hydroxyl group of the Y202 is partially transferred to the S37 hydroxyl oxygen with the distances of 1.24 Å and 1.19 Å between the proton and the oxygens of Y202 and S37, respectively. The distance between the proton and the amino nitrogen is 1.09 Å indicating that this proton is not transferred to Y202 in the TS state. The bond between the nitrogen atom of the amino group and the carbon of the carbonyl group is partially formed with the distances of 1.61 Å and 2.04 Å between the carbon and the nitrogen and the carbon and the hydroxyl oxygen of S37, respectively.

The angles formed by the amino N atom are 110.4°, 108.1°, 109.3°, 106.3°, 106.0°, and 116.6° implicated with the two hydrogens, and between the CA-N-C atoms. All angles are close to the ideal 109.5°, indicating that, as opposed to the mechanism with the direct proton transfer, the TS geometry in the mechanism with the participation of Y202 is devoid of steric hindrance with the structure close to the ideal tetrahedral geometry, which explains the much lower activation barrier for this mechanism. To quantify the effect of the enzyme environment we computed the free energy of the TS state in vacuum and in the COSMO implicit solvent model using the geometry of the QM region taken from the protein simulations. In vacuum and solvent, the energy of the transition state is 4.2 and 1.9 kcal/mol higher than in the enzyme environment, respectively. Thus, in contrast to mechanism I, the enzyme environment favors the reaction of the mechanism II with the participation of Y202. This also argues in favor of mechanism II.

It is easier to take the proton from the hydroxyl group of the phenol than from other alcohols. The anomalously low pK_a_ of phenol relative to other alcohols is explained by the particular structure of the aromatic ring. To test if other amino acids could accomplish a similar role in the catalytic reactions, we performed calculations with a part of the QM region taken from the protein simulations. The QM region contained the sidechain of S37 including Cα, the dipeptide, and the hydroxyl group of Y202 and the Cζ atom of Y202. Three additional protons were added to Cζ to form a methanol molecule. Thus, this should serve as a model for the reactions with the proton transfer accomplished by other amino acids containing a hydroxyl group, such as serine and threonine, but lacking an aromatic ring. The three hydrogens of the methyl group of the methanol molecule were energetically optimized at the DFT level of theory for each representative structure. The computed free energy of TS in solvent using the COSMO implicit solvent model is 3.3 kcal/mol higher than in the calculations with the tyrosine side chain. Thus, the hydroxyl group of tyrosine is better suited for the proton transfer.

### The role of the residue E182

**Figure 4:**
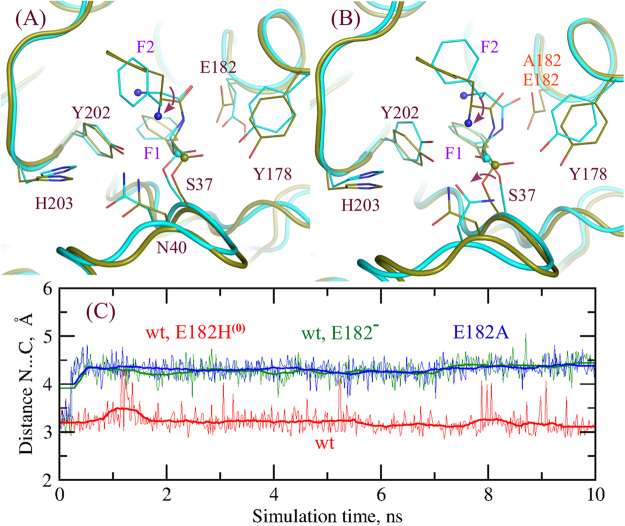
Effect of the protonation or mutation of the residue E182 on the geometry of the dipeptidyl-enzyme. (**A**) Snapshots from the MD simulations of the wild-type AlbC with the protonated form and the deprotonated form of E182 shown in dark green and cyan, respectively; (**B**) snapshots from the MD simulations of the wild-type AlbC with the protonated E182 and E182A mutant shown in dark green and cyan, respectively; conformational changes associated with the E182A mutation or the protonation state are indicated by the red arrows; (**C**) the distance between the N and C atoms of the dipeptide, shown as spheres in panels (**A**) and (**B**), during the first 10 ns MD simulations.

Residue E182 was suggested to play a key role in different steps of the catalytic circle of AlbC. It was proposed that E182 fulfills the role of the general base in the formation of the diphenylalanyl enzyme intermediate. In this step E182 would deprotonate the ammonium group of the phenylalanyl enzyme intermediate to form a nucleophile that subsequently attacks the incoming carbonyl of the second Phe-tRNA^Phe^ to form the diphenylalanyl enzyme intermediate. In the diphenylalanyl intermediate E182 must be in the protonated form and has been suggested to stabilize the backbone conformation of the diphenylalanyl intermediate required for the subsequent cyclization step^7^. We computed the deprotonation free energy of E182 using the PB/LRA method to be 8.9 ± 0.6 kcal/mol. In combination with the pK_a_ value of the glutamic acid in solvent of 4.4, the pK_a_ value of E182 of AlbC in the diphenylalanyl enzyme intermediate is 10.8 pK_a_ units. This is in an agreement with the pK_a_ prediction of 9.9 and 11.4 using the empirical PROPKA method and the crystal structure with the ZPK ligand (PDB reference code 4Q24)^7^ and with the modeled dipeptide, respectively. Thus, calculations in the agreement with the crystal structure predict that E182 should be protonated in the complex. The unusual protonation state of E182 can be rationalized by the fact that E182 is positioned deeply in the hydrophobic pocket formed by residues F186, Y178, V179, M152, V156, V67, and the Phe2 sidechains of the diphenylalanyl group. When E182 is deprotonated and charged the interaction between one of oxygens of E182 and the NH group of the dipeptide is maintained, while E182 rotates and three water molecules enter the hydrophobic cavity to interact with the other oxygen of E182, shown in Fig. 4.

To further test the role of E182 we performed MD simulations with the E182A variant of AlbC. With A182 the backbone of the dipeptide departs from its crystal position within the first nanosecond of the MD simulations, so that the distance between the amino group and the carbon atom of the carbonyl group attached to the S37 increases to 4.2 Å. Figure 4 shows the structures of the dipeptidyl-enzyme in the simulations with the wild-type protein and with the E182A variant as well as the distance between the amino N atom and the C atom of the backbone group of the phenylalanine of the dipeptide covalently attached to S37. Thus, in this orientation the N-terminus group cannot react to form the cyclodipeptide. Similarly, in the case when E182 is deprotonated the dipeptide backbone rotates so that the amino group is not poised for the reaction as shown in Fig. 4. Overall, interactions with the neutral form of E182 are needed to maintain the correct orientation of the dipeptide needed for the cyclization reaction. To test the direct effect of E182 on the catalytic reaction, we computed the free energy of the structures along the Nudged Elastic Band (NEB) path but with the zero charges on the atoms of the sidechain E182 up to Cγ. With zero charges on E182 the barrier for the cyclization reaction slightly decreases by 0.9 kcal/mol and the free energy of the product increases by 0.6 kcal/mol relative to the reactant structure. The direct role of E182 in the catalytic reaction of cyclization is small, but, as reasoned above, it has an important role in maintaining the correct geometry of the reacting groups. The important role of E182 is also consistent with its strict conservation among the entire CDPS family^1^.

### Role of the residues Y178, N40 and H203

The conserved residue Y178 was shown to play a significant role in the transfer of the first phenylalanine from the Phe-tRNA^Phe^ to S37 of AlbC. It was established that the Y178F substitution substantially impairs both enzymatic activity and the formation of the phenylalanyl enzyme intermediate in the first step of the reaction^5^. To study the direct effect of Y178 on the cyclization step of the reaction we computed the free energies with the Y178F variant using the structures computed with the wild-type protein. The activation barrier for the cyclization step with the Y178F mutation is 0.9 kcal/mol lower than the free energy barrier computed for the wild-type protein. The product is also lower relative to the reactant state by 1.3 kcal/mol. Thus, the hydroxyl group of Y178 does not have a significant direct effect on the cyclization step. To study the role of Y178 on the geometry of the catalytic center and the dipeptide in particular, we performed MD simulations of the Y178F variant of AlbC. The superposition of structures from the MD simulations with the wild-type protein and the Y178F variant taken after 30 ns are shown in Fig. SI-1. During simulations with the Y178F variant two water molecules from the bulk solvent substituted interactions between Y178 and the carbonyl group of the dipeptide Phe1. The ester group of the dipeptidyl group flips in the course of MD simulations with the Y178F variant. The distance between the N and C atoms of the dipeptide also increases. In this conformation Y202 cannot participate in the proton transfer, which is in line with the experimental observation that the Y178F change diminishes the catalytic efficiency of the enzyme.

**Figure 5:**
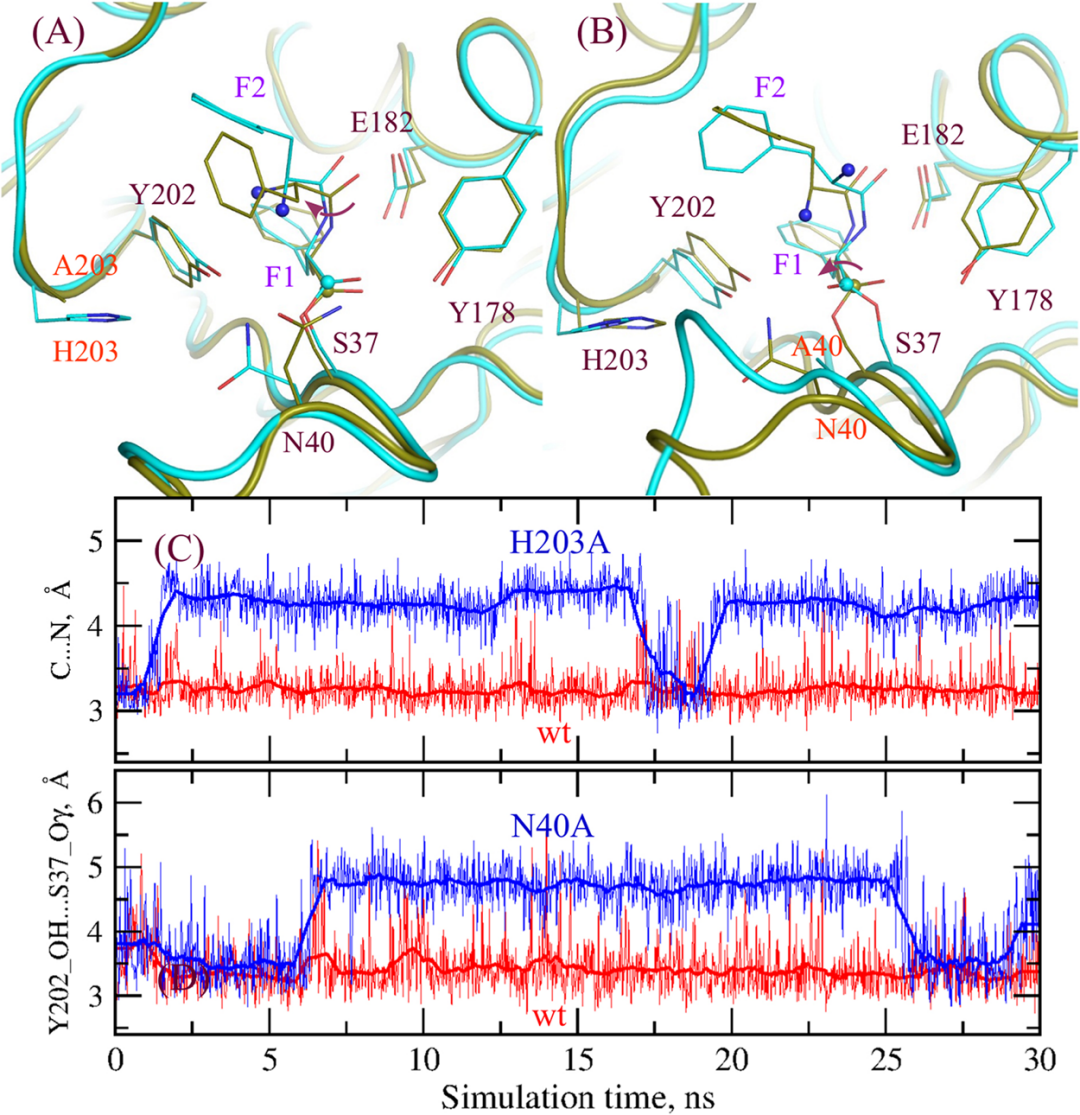
Effect of mutation of residues H203 and N40 on the geometry of the dipeptidyl-enzyme. (**A**) Snapshots from the MD simulations of the wild-type AlbC and H203A variant shown in dark green and cyan, respectively; (**B**) snapshots from the MD simulations of the wild-type AlbC and N40A variant shown in dark green and cyan, respectively; conformational changes associated with the H203A or N40A mutations are indicated by the red arrows; (**C**) the distance between the N and C atoms of the dipeptide in the wild-type AlbC and H203A simulations; (**D**) the distance between the hydroxyl oxygen of Y202 and the dipeptide carbonyl oxygen in the simulations with the wild-type AlbC and N40A variant.

Both N40 and H203 were identified to be important for the reaction catalyzed by AlbC. Mutations of either of these residues into alanine strongly impairs catalytic activity^7^. To test the effect of these residues on the cyclization step we performed unrestrained MD simulations of the variants N40A and H203A. Structures are shown in Fig. 5 along with the important distances in the MD simulations. With the H203A variant the dipeptidyl group changes conformation very similar to the E182A mutation leading the non-reactive conformation with the amino group positioned far from the C atom of the dipeptide. In the simulations with the N40A variant the ester group of the dipeptide, which is covalently attached to S37 flips after 6 ns of MD simulations. In this conformation Y202 cannot transfer the proton since it hydrogen bonds the carbonyl oxygen and is also distant from the hydroxyl group of S37. H203 and N40 were found to make a water mediated interaction in the crystal structure^7^. The alanine substitution of either of these residues has an effect on the conformation of the other residue and the important loop, CL1, as shown in Figs 1 and 5. In turn, this affects the conformation of the dipeptide, which is covalently attached to the S37 also belonging to CL1. Thus, residues H203 and N40 work in pairs, suggesting that there is only a limited set of residue pairs compatible with the catalytic function. This result is directly corroborated by the existence of two subfamilies of CDPS enzymes, one having H203 and N40 and the other family having P203 (in AlbC numbering) with a non-conserved residue found at the equivalent of position 40 of AlbC^1,13^. Overall, Y178, N40 and H203 are important residues needed to maintain the correct reactive conformation of the dipeptidyl group.

## Discussions

In the present work, we have employed ab initio QM/MM hybrid potential methods to investigate possible mechanisms of the cyclization reaction catalyzed by the AlbC synthase of the CDPS family. This work was motivated by the recent experimental crystallographic study, which solved for the first time a CDPS in the dipeptidyl-enzyme state^7^. The cyclization reaction requires nucleophilic attack of the N atom of Phe2 on the C atom of the dipeptide, and so requires a general base to abstract a proton from the amino group. On the other hand, when the cyclodipeptide is formed the hydroxyl group of the residue S37 should be protonated, since the experimental pK_a_ of the serine hydroxyl group in solvent is about 16 (the pK_a_ of ethanol)^12^. The lack of any general acid in the vicinity of S37 suggests that the proton from the NH_2_ group should be transferred to the hydroxyl group of S37 during the reaction in a concerted manner. In this work we considered three mechanisms for the cyclization reaction. The first reaction involves Y202 in a deprotonated form as a general base. Protonation free energy calculations show that Y202 strongly prefers to be in the neutral protonated form, and thus, the mechanism with Y202 as a general base is unlikely. The hydrophobic environment of AlbC helps stabilize the neutral state of Y202. Overall, it appears that the function of the residues of AlbC is to desolvate the catalytic groups to stabilize the protonation state and geometry needed for the catalytic function. Thus, the protein at the dipeptidyl intermediate state is pre-organized for the cyclization reaction.

We also tested a mechanism with the direct proton transfer from the amino group to S37, which is simultaneous with the attack of the N atom on the ester carbon. The computed free energy barrier for this reaction mechanism is very high, 61.6 kcal/mol, which is explained by steric hindrance in the transition state caused by the transition state geometry being far from the ideal tetrahedral geometry. Finally, we modeled a mechanism involving Y202 as a proton relay. The computed free energy of the transition state is much lower, 12.4 kcal/mol, than the free energy computed for the mechanism with the direct proton transfer. The geometry of the transition state is very close to the ideal tetrahedral electron pair geometry explaining the much lower free energy barrier for this mechanism. The results also show that the hydroxyl group of tyrosine is better suited to serve as a proton relay in the reaction than hydroxyl groups of the amino acids due to the peculiar structure of the benzene ring, which also explains the strict conservation of Y202.

Finally, we note that this mechanism discovered in this work can be universal among other CDPSs, since it relies on the amino groups conserved among the entire CDPS family. Indeed, the catalytic residues S37 and Y202, involved in the formation of the aminoacyl enzyme and cyclization of the dipeptide respectively, are strictly conserved in all active CDPSs. The Y178 and E182 residues, responsible for anchoring the aminoacyl moiety of the first substrate and formation of the dipeptidyl enzyme are also well conserved.

## Methods

### Molecular dynamics simulations

The crystal structure of the CDPS synthase was obtained from the Protein Data Bank (PDB), entry 4Q24^7^. The structure corresponds to the S37C variant of AlbC, which is from organism *Streptomyces noursei* and synthesizes cyclo(L-Phe-L-Leu) and cyclo(L-Phe-L-Phe). The crystal structure contains a ligand, N-carbobenzyloxy-L-Phe-methyl ketone (ZPK) covalently attached to C37. The mutation S37C was introduced to attach the ZPK substrate analog to the enzyme. The native diphenylalanyl dipeptide intermediate of AlbC was built from the existing ZPK ligand by converting the S*γ* sulfur of C37 into an oxygen; deleting the C91 atom and making a covalent bond between the O atom of residue 37 and the C92 atom for the dipeptide; converting atom O11 into C-atom; finally, the amino group of the dipeptide was built through ideal stereochemistry.

The molecular dynamics simulations included all protein residues and the dipeptidyl group attached to S37. Protonation states of histidines were assigned by visual inspection and ideal stereochemistry; protonation states of other residues were assigned using PROPKA^14,15^. Thus, residue H203 near the catalytic site was set to be neutral and protonated on the N*ε* nitrogen based on the short distance of 2.83 Å between the N*δ* atom of H203 and its backbone amide nitrogen indicating that the N*δ* atom should be deprotonated. Glutamate 182 was protonated as indicated by the short distance of 2.73 Å between the O*ε* oxygen of E182 and the backbone O atom of Phe2 of the dipeptide in the crystal structure.

In addition to crystal waters, a 78-Å cubic box of water was overlaid, and waters overlapping the protein, the dipeptidyl group and crystal water molecules were removed. Periodic boundary conditions were assumed; i.e. the entire 78-Å box was replicated periodically in all directions. All long range electrostatic interactions were computed efficiently by the particle mesh Ewald method^16^, and the appropriate number of potassium counterions were included to render the system electrically neutral. To prevent drift of the protein, the center of mass of the protein Cα atoms was weakly restrained to the origin of the system by a harmonic potential with a force constant of 10 kcal/mol/Å^2^. MD simulations were performed at constant room temperature and pressure, after 200 ps of thermalization. The CHARMM27 force field^17,18^ was used for the protein and the modified version of the TIP3P water model^17,19,20^. Calculations were done with the NAMD program^21^. To simulate the effect of single-point mutations of the residues, E182A, Y178A, N40A and H203A that are in the direct vicinity of the dipeptide, the sidechain of the mutated residue was deleted in the structure for the wild-type protein up to the Cβ atom and the Cβ hydrogens were built. Thirty nanoseconds of unrestrained molecular dynamics were performed with each of wild-type and mutant proteins.

### Hybrid potential simulations

For the hybrid potential QM/MM calculations, the AlbC protein was partitioned between QM and MM regions. The QM region had 42 atoms, comprising the backbone atoms of the dipeptide covalently attached to S37 and the sidechains of Y202 and S37. Hydrogen link atoms were used to treat the covalent bonds that pass through the border between the QM and MM regions. The additive QM/MM coupling scheme was adopted as in the previous studies with electrostatic embedding of the QM region^22^. The hybrid potential simulations included protein residues within a 24 Å sphere, centered on the dipeptide binding site. Throughout the molecular simulations, protein atoms between 20 and 24 Å from the sphere’s center were harmonically restrained to their experimental positions as in the previous studies^23–26^. The MM region had around 44700 atoms. The QM region was electrically neutral.

The atoms in the MM region were represented with the CHARMM27 force field^17,18^ whereas a density functional theory (DFT) method was used for the QM region with the TPSS functional^27^ and the Ahlrich’s def2-SVP AO basis set^28^. The widely-used TPSS functional was demonstrated to reproduce accurately the transition state structures and energies^29,30^. Single point calculations on the optimized structures were done with the larger def2-TZVP basis^31^ which has polarization functions on all atoms. In all calculations the RIJCOSX algorithm for the two-electron integrals was employed for efficient calculations as implemented in the ORCA program^32,33^. The algorithm treats the Coulomb term via a RI approximation and the exchange term via semi-numerical integration. We used the D3 London dispersion correction in the Becke-Johnson sampling scheme (indicated by “−D3” appended to the functional name)^34^. All geometry optimizations and reaction path calculations were performed with the QM(DFT)/MM potential.

The starting coordinates of the complexes were taken after 30 ns of MD simulations on the systems described in the section Molecular Dynamics Simulations. The QM(DFT)/MM calculations were performed with the pDynamo software^22^ and its interface to the ORCA program^35^. No truncation or cut-off was employed to calculate non-bonding interactions. The reaction path was optimized with the new HEBS method implemented in pDynamo^36^, which is a chain-of-states method and a hybrid of the nudged elastic method and the string method. In the HEBS method 20 replicas were used to represent the reaction pathway, which we found sufficient enough for a set of reactions in enzymatic systems in the previous extensive study^36^. After a regular HEBS calculation was converged, the image with the highest energy in the path was optimized using the climbing image method (CI-NEB). We showed in the previous study that application of the CI-NEB is required to fully refine saddle point structures^36^.

### QM/MM reaction path free-energy perturbation (QM/MM-FEP) calculations

We calculated free energies of the structures along the HEBS pathways using the free energy perturbation (FEP) method of Kastner *et al*.^37^, which is also similar to that of Zhang and co-workers^38^. The system setup was similar to that described in section Molecular Dynamics Simulations, except that the atoms in the QM region and the link atoms were constrained at their corresponding optimized geometries from the HEBS profile. CHELP charges^39^, computed with the TPSS/def2-TZVP DFT-D3 method, were used for the atoms in the QM region and CHARMM27 charges for those in the MM region.

MD simulations were performed at constant room temperature and pressure for 1 nanosecond for each replica of the HEBS profiles giving 20 ns of the simulation time. The free-energy change between images *i* and *i* + 1 due to the QM/MM interactions was calculated according to the formula:

1 $${{\rm{\Delta }}{\rm{A}}}^{i\to i+1}=-1/\beta ln{\langle exp(-\beta {\rm{\Delta }}{E}_{pert}^{i\to i+1})\rangle }_{mm,i},$$

where ${\rm{\Delta }}{E}_{pert}^{i\to i+1}$ is the perturbation energy computed with the coordinates of the MM atoms from the MD simulation of image *i*, and the QM atom positions of image *i* + 1. Estimates of the free energies of the atoms in the QM region were obtained using a rigid-rotor harmonic-oscillator approximation after normal mode analysis^24^. However, we found that these contributions to the calculated free energy differences between the different structures were negligible^24^.

### Poisson-boltzmann linear response approximation (PBLRA)

To determine the protonation free energy of Y202 and the N-terminus end group of the dipeptidyl group, in the AlbC protein and alone in solution, we used a Poisson-Boltzmann Linear Response Approximation (PB/LRA)^40^. Protonations of the hydroxyl group of Y202 and the amino group of the dipeptide were modeled by changing selected atomic charges. The corresponding free energy change was computed both in the protein complex and for the model compound in solution, using conformations taken from 30 ns MD simulations. The free energy was approximated by the continuum electrostatic free energy, in which the protein and dipeptide atoms are explicitly included in the calculation but solvent and counterions are replaced by a dielectric continuum. The free energy change in either medium (protein, solvent) can be written as:

2 $${\rm{\Delta }}G=\sum \delta {q}_{i}({\langle {V}_{i}\rangle }_{A}+{\langle {V}_{i}\rangle }_{B})/2,$$

where the sum is over all the atoms of the titratable group; *δq*_*i*_ is the difference in the atomic charge as a result of the protonation; and 〈*V*_*i*_〉 is the electrostatic potential on atom *i* averaged over simulations with the protonated and deprotonated group (state A and B). Averaging was performed over the ensemble of conformations drawn from an MD simulation when the hydroxyl group is protonated or deprotonated (250 snapshots taken from the 30 ns MD simulations). The electrostatic potentials were computed for each MD conformation by numerically solving the Poisson-Boltzmann equation of continuum electrostatics, where the protein and substrate were treated as a single dielectric medium with a dielectric constant of 2; solvent was treated as another medium with a dielectric constant of 80, the experimental value for bulk water. A low dielectric value was found to be appropriate for the protein as the conformational changes induced by the proton binding are explicitly modeled by simulating its protonated and deprotonated states^40,41^.

The boundary between the two dielectric media was defined as the protein/ligand molecular surface, computed with a 1.4 Å radius of the probe sphere. The system was discretized using a spacing of 0.4 Å. The linearized Poisson-Boltzmann equation was solved using the PBEQ module^42^ of the CHARMM program^43^ with Coulombic boundary conditions. An ionic strength corresponding to a 0.15 M concentration of monovalent ions was used. The same procedure was followed for tyrosine in water, using the conformations drawn from the protein simulations. The pK_a_ shift due to the protein environment has the form:

3 $$p{K}_{a}^{prot}-p{K}_{a}^{solv}=({\rm{\Delta }}{G}^{prot}-{\rm{\Delta }}{G}^{solv})/2.303RT,$$

where $p{K}_{a}^{prot}$ and $p{K}_{a}^{solv}$ are *pK*_*a*_’s of a titratable group in protein and solvent respectively; Δ*G*^*prot*^ and Δ*G*^*solv*^ are the free energy change in protein and solvent respectively; *R* is the molar gas constant and *T* is the temperature.

## Electronic supplementary material

Supplementary Information
