# Supplementary material for: Cyclization Reaction Catalyzed by Cyclodipeptide Synthases Relies on a Conserved Tyrosine Residue
Source: Sci Rep. 2018 May 4;8:7031. doi: 10.1038/s41598-018-25479-5 (PMC5935735; doi:10.1038/s41598-018-25479-5)
Supplement: Supplementary file 1 — Supplementary Information [file 41598_2018_25479_MOESM1_ESM.pdf]

Supplementary Information

For

# Cyclization Reaction Catalyzed by Cyclodipeptide Synthases Relies on a Conserved Tyrosine Residue

Emmanuelle Schmitt, Gabrielle Bourgeois, Muriel Gondry and Alexey Aleksandrov

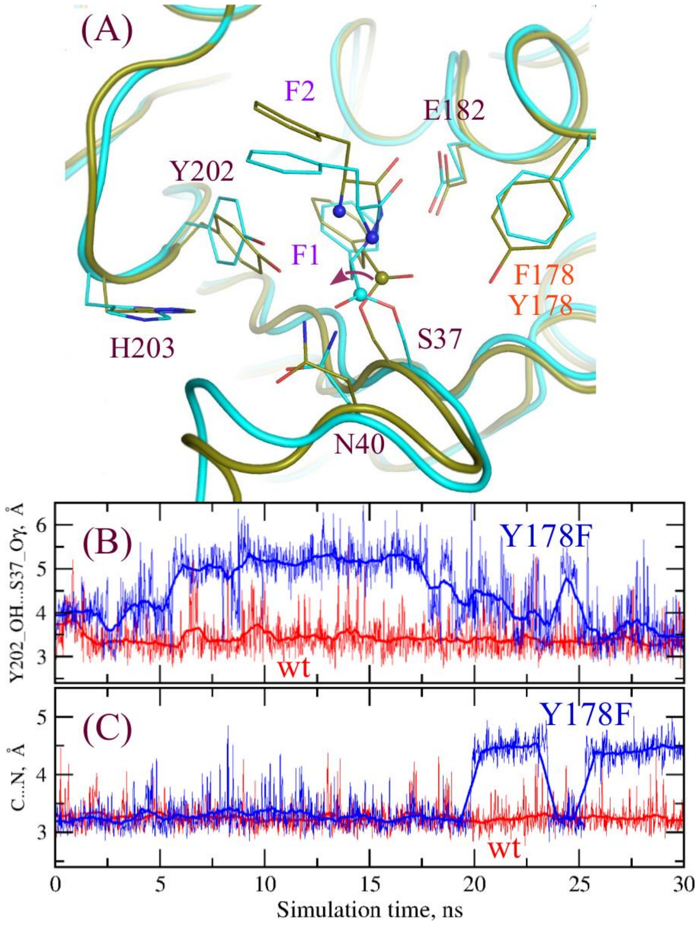


**Figure SI-1.** Effect of mutation Y178F on the geometry of the dipeptidyl-enzyme. (A) Snapshots from the MD simulations of the wild-type AlbC and Y178F mutant shown in dark green and cyan, respectively; (B) the distance between the hydroxyl oxygen of Y202 and the S37 hydroxyl oxygen in the simulations with the wild-type AlbC and Y178F mutant; (C) the distance between the N and C atoms of the dipeptide in the wild-type AlbC and Y178F simulations.
